# Low penetrance of a *SDHB *mutation in a large Dutch paraganglioma family

**DOI:** 10.1186/1471-2350-11-92

**Published:** 2010-06-11

**Authors:** Frederik J Hes, Marjan M Weiss, Sanne A Woortman, Noel F de Miranda, Patrick A van Bunderen, Bert A Bonsing, Marcel PM Stokkel, Hans Morreau, Johannes A Romijn, Jeroen C Jansen, Annette HJT Vriends, Jean-Pierre L Bayley, Eleonora PM Corssmit

**Affiliations:** 1 Departments of Clinical Genetics, Leiden University Medical Center, POBox 9600, 2300 RC Leiden, The Netherlands; 2 Pathology, Leiden University Medical Center, POBox 9600, 2300 RC Leiden, The Netherlands; 3 Surgery, Leiden University Medical Center, POBox 9600, 2300 RC Leiden, The Netherlands; 4 Nuclear Medicine, Leiden University Medical Center, POBox 9600, 2300 RC Leiden, The Netherlands; 5 Endocrinology, Leiden University Medical Center, POBox 9600, 2300 RC Leiden, The Netherlands; 6 Otorhinolaryngology, Leiden University Medical Center, POBox 9600, 2300 RC Leiden, The Netherlands; 7 Human Genetics, Leiden University Medical Center, POBox 9600, 2300 RC Leiden, The Netherlands

## Abstract

**Background:**

Germline mutations of the succinate dehydrogenase subunit B gene (*SDHB*) predispose carriers for paragangliomas, and current estimates of the chance of mutation carriers actually developing tumors (penetrance) are high. We evaluate the phenotype and penetrance of a germline *SDHB *mutation in a large and clinically well-characterized paraganglioma family.

**Methods:**

Following identification of the mutation in a 31 year old index-patient, extensive clinical screening was performed in mutation carriers to evaluate the presence of head and neck, thoracic and abdominal paragangliomas. Presymptomatic DNA testing was performed in 19 family members.

**Results:**

DNA analysis detected 14 further *SDHB *mutation carriers. Three mutation carriers (median age 78 years) declined clinical surveillance, but had no clinical signs or symptoms associated with paragangliomas. The remaining 11 mutation carriers (mean age 53, range 37-76 years) consented to clinical screening. In only two, aged 43 and 48 years, were subclinical vagal paragangliomas identified.

**Conclusions:**

Only three of the fifteen mutation carriers in this family have developed paraganglioma, which results in a calculated penetrance of 26% at 48 years of age. This figure is lower than current estimates, and we conclude that the co-operation of this family allowed an almost complete attainment of mutation carriers, and the extensive clinical evaluation carried out allowed us to identify all affected individuals.

## Background

Germline mutations in the succinate dehydrogenase (SDH) subunit genes *SDHB*, *SDHC*, *SDHD *and *SDHAF2 *predispose carriers to tumors of the paraganglia, the paragangliomas [1]. Parasympathetic paragangliomas arise in the head and neck, whereas sympathetic paragangliomas are generally intrathoracic, retroperitoneal or located in the adrenal medulla (pheochromocytoma). Mutations of *SDHD *are the most prominent cause of head-and-neck paragangliomas, whereas mutations in *SDHB *frequently result in adrenal and extra-adrenal paragangliomas, and related malignant disease [2-6].

Germline *SDHB *mutations show an autosomal dominant inheritance pattern, but the full clinical implications of *SDHB *mutations have remained obscure since most studies to date have described predominantly affected index cases [3,4]. *SDHB *germline mutation carriers often present as apparently sporadic patients, i.e. with a negative family history for paragangliomas. There is no evidence that these patients are genuine *de novo *cases and when studied, other mutation carriers can usually be identified in the family. While it is recognized that the penetrance of *SDHB *mutations is incomplete and age dependent [1,3,4], particularly in comparison with *SDHD*, the current estimates are likely to be inflated because many mutation carriers remain asymptomatic. Only two other large families has been described in which presymptomatic family members were identified by genetic screening [7,8].

The aim of the current study was to assess the phenotype and penetrance of a germline *SDHB *splice-site mutation (c.423+1G > A, intron 4) in a large Dutch paraganglioma family. This family was identified following DNA analysis in an apparently sporadic index-patient with an extra-adrenal paraganglioma. We carried a full clinical and genetic assessment in all consenting family members.

## Methods

Informed consent was obtained for DNA testing according to protocols approved by the local ethics review board. Mutation carriers were referred to the outpatient clinic of the departments of Endocrinology and Otorhinolaryngology of the Leiden University Medical Center, which is a tertiary referral center for paragangliomas. All patients were included in a previously described standardized follow-up protocol [6,9,10] and gave permission for publication of clinical test results. Briefly, *SDHB *mutation carriers were seen at least yearly, and catecholamine screening was carried out in duplicate 24 hours urine under strict dietary medication regulations.

Epinephrine, norepinephrine, metanephrine, normetanephrine, dopamine, VMA and 3-methoxy-tyramine excretion in 24 h urine were quantified as described [6,9,10]. Reference ranges were: epinephrine < 0.16 μmol/24 h, norepinephrine 0.06-0.47 μmol/24 h, metanephrine 33-90 μmol per mol creatinine, normetanephrine 64-260 μmol per mol creatinine, dopamine 0.46-3.40 μmol/24 h, VMA < 30 μmol/24 h, and 3-methoxy-tyramine 45-197 μmol per mol creatinine.

In cases of excessive secretion, MIBG scanning and additional whole-body MRI and/or CT imaging were performed. Whole-body MRI and/or CT imaging are performed at least every two years regardless of catecholamine levels. The head and neck region were examined annually by an Ear-Nose-Throat (ENT) - surgeon and all head and neck paragangliomas were assessed yearly by MRI. Presymptomatic mutation carriers have an MRI of the head and neck region every 3-5 years.

### DNA analysis and haplotyping

Sequence analysis of the coding region including the splice sites of the SDHB gene was performed according to standard procedures (details available upon request), using the NT_004610.18 reference sequence [11]. The mutation was described following the nomenclature guidelines of the Human Genome Variation Society http://www.hgvs.org/. Haplotyping was performed following standard procedures (details available upon request) with the following markers: D1S436, D1S2697, D1S170, D1S3669, D1S2826 and D1S2644. The distance between the last and the first marker (D1S436 and D1S2644) is ~ 3.35 Mb.

### Statistics

Penetrance was calculated using the reverse Kaplan-Meier method. Kaplan-Meier survival tables included all mutation carriers, including the three aged individuals who declined screening. 95% confidence intervals are also depicted.

## Competing interests

The authors have disclosed any affiliation with any organization with a financial interest, direct or indirect, in the subject matter or materials discussed that may affect the conduct or reporting of the work submitted.

## Results

**Figure 1:**
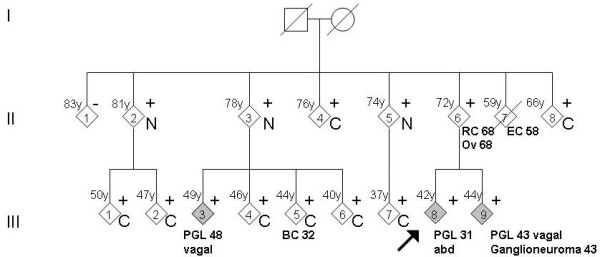
**Pedigree with clinical data**. The arrow shows the index patient. The non-carriers have been collapsed to minimize recognition of the pedigree.+, mutation present; -, mutation absent; C, clinical screening; N, no symptoms (but not screened); RC, rectal carcinoma; Ov, ovarian carcinoma; EC, endometrial carcinoma; BC, breast cancer (tumors with age at diagnosis in years).

The index patient, a 31-year old man, was referred for evaluation of hypertension (III-8, figure 1). His unmedicated blood pressure was 165/105 mmHg, without paroxysmal episodes. Twenty four-hour urine analysis showed repeatedly increased excretion of VMA (58-80 μmol/24 hr) and norepinephrine (3.86-7.29 μmol/24 hr). ENT examination and MRI produced no evidence of head and neck paraganglioma. Abdominal MRI showed a 3 cm mass between the aorta and inferior vena cava, negative by ^l23^I-MIBG-scan. Following preoperative alpha- and betablockade, the mass was resected and the histological diagnosis was paraganglioma. After removal of the tumor, blood pressure and catecholamines normalized.

The patient's family history was negative for paraganglioma, but DNA analysis revealed a mutation in the *SDHB *gene, c.423+1 G > A, a splice-site mutation previously identified and shown by us to lead to mis-splicing [11]. This mutation has subsequently been identified in other paraganglioma patients and is known to be pathogenic.

Subsequently, 19 family members of the index patient consented in presymptomatic mutation screening. Five non-carriers and 14 carriers were identified, of whom 11 consented to clinical screening. The remaining three mutation carriers were of a median age of 78 years (II-2,3,5), declined clinical surveillance, but had no clinical symptoms to suggest paraganglioma. Screening resulted in the identification of two family members (III-9 and III-3) with subclinical vagal paragangliomas.

The sibling of the index patient (III-9), a 42-year old man, had no clinical indication of paraganglioma and no suspicious symptoms, with lightly elevated blood pressure (140/85 mmHg), but MRI of the neck region showed a right-sided vagal paraganglioma. Analysis of 24-hour urine showed a slightly increased excretion of VMA (34 μmol/24 hours). MIBG- and MRI-scans of the abdomen showed two small lesions (diameter 5 mm) with increased uptake on MIBG, possibly para-aortic paragangliomas. The lesions were laparoscopically removed and the histopathological diagnosis was ganglioneuroma. Postoperatively, urinary catecholamines normalized.

Patient (III-3), an asymptomatic 48-year old male cousin of the index-patient, had recent hypertension (140/85 mmHg). An MRI of the head-and-neck area showed a right-sided vagal paraganglioma. 24-hour urine analysis revealed increased excretion of the dopamine metabolite 3M-tyramine (688 and 938 μmol per mol creatinine), without increased excretion of other catecholamines. Abdominal and mediastinal MRI showed no paraganglioma.

In addition to the described patients (III-3,8,9), the remaining mutation carriers underwent clinical evaluation for paragangliomas. These subjects had a mean age of 53 years (range 37-76 years), and despite full body imaging and extensive biochemical analyses, no indications for the presence of paragangliomas or pheochromocytomas could be found. It is worth noting that including the three symptom-free carriers who declined screening, six mutation carriers aged 66-81 years were negative by clinical screening or by lack of any manifestations of paragangliomas.

**Figure 2:**
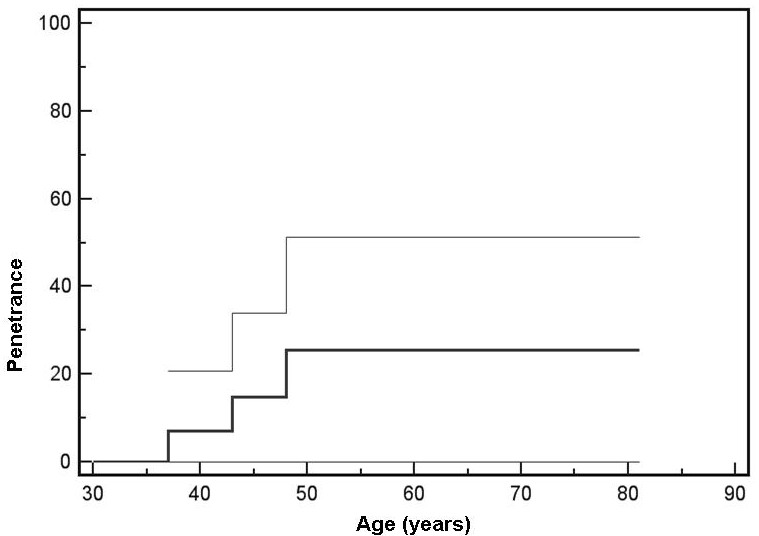
**Kaplan-Meier curve of age-related penetrance of *SDHB *c.423+1G > A carriers**. Thin lines represent 95% confidence intervals.

Kaplan-Meier analysis of the mutation cases showed a penetrance of 26% at 48 years for the germline SDHB c.423+1G > A mutation, indicating a remarkable reduced penetrance (Figure 2).

### Additional SDHB c.423+1G > A mutation carriers

In addition to the family described above, four Dutch paraganglioma patients were found to be carriers of the c.423+1G > A mutation. Since these patients were not known to be related to the family, we carried out microsatellite marker analysis to determine if the mutation was located on a shared haplotype, which would indicate a (distant) family relationship. Founder mutations are well known in the Dutch population and the *SDHD *D92Y mutation is a striking example [12]. As expected, a shared haplotype was found in the family, and the same haplotype was also found in all four sporadic patients. This marker haplotype has a frequency of less than 1% in the Dutch population and the likelihood of five unrelated cases carrying this haplotype is > 1.1 ×10^-10^, indicating that all patients are related.

## Discussion

This study describes a large SDHB-related family, in which an extensive and detailed clinical and genetic screening program has been carried out. The identification of a relatively large number of presymptomatic mutation carriers provides evidence for a lower penetrance of an *SDHB *mutation than suggested in previous studies. In addition, haplotype analysis in four apparently sporadic patients demonstrated that these cases are related to the described family, again reinforcing the previously only suggestive evidence that behind every paraganglioma patient with an SDHB mutation, many asymptomatic mutation carriers remain under the clinical radar.

Our observation that only three of fifteen *SDHB *mutation carriers developed paragangliomas indicates a penetrance of only 26% at 48 years, is lower than previous studies (50% - 77% at 50 years) [3,4]. The fact that *SDHB *mutation carriers often present as apparently non-familial cases was suggestive of reduced penetrance [4,13-15], and even founder mutations of *SDHB *have presented initially in isolated cases or families [16,17].

Any accurate estimation of penetrance requires full clinical screening of as many genetically affected family members of index cases as will co-operate. Even under such ideal scenarios, the possibility of bias remains in that unaffected family members are less likely to be inclined to undergo extensive clinical investigation. The estimation of penetrance based on index cases, as is commonly practiced, is tempting but erroneous, and studies that include only a limited number of additional family members will also only provide an approximation to the true penetrance. Initial estimates of penetrance are often inflated, and tend to decline as more extensive families are evaluated. Our study once again reiterates that only well-conducted family studies can be used to assess penetrance.

The only other extensive study carried out to date on the penetrance of SDHB, by Solis et al., reported a penetrance of 35% at 50 yrs [7]. This estimate is higher than that reported here but may also be upwardly biased because 18 mutation carriers declined clinical screening, and these may have been individuals who felt no pressing need to undergo screening. It is worth noting that Solis et al. were able to diagnose 11 paraganglioma patients amongst 41 mutation carriers (27%).

An obvious caveat of our study is the size of the family and the low number of affected patients, reflected in the wide confidence intervals of the Kaplan-Meier analysis, and also the current age of some mutation carriers. In addition, a possible mutation specific genotype-phenotype effect may exist with some normal transcript being produced from the mutated transcript, although this is unlikely in light of the importance of the consensus splice donor site. Another, although less probable caveat, is that of a possible family-specific modifier of penetrance. The further elucidation of the true penetrance of *SDHB *mutations requires the description of many more families in a manner similar to that described here.

## Conclusions

In summary, we described a large and extensively screened family with a germline *SDHB *mutation. Thorough analysis of large cohorts of both symptomatic and presymptomatically detected mutation carriers and haplotyping are required to further elucidate the natural history of SDH-associated disease.

## Abbreviations

(SDHB): succinate dehydrogenase subunit B gene; (VMA): vanillylmandelic acid.

## Competing interests

The authors declare that they have no competing interests.

## Authors' contributions

All authors were involved in drafting the article or revising it critically for important intellectual content, and all authors approved the final version to be published. Study conception and design: FJH, JPLB and EPMC. Acquisition of data: NFdM, SAW, PvB, BAB, MPMS, JCJ. Analysis and interpretation of data: FJH, JPB, HM, JAR, AHJTV.

## Pre-publication history

The pre-publication history for this paper can be accessed here:

http://www.biomedcentral.com/1471-2350/11/92/prepub
